# Mutant p53^K120R^ expression enables a partial capacity to modulate metabolism

**DOI:** 10.3389/fgene.2022.974662

**Published:** 2022-09-26

**Authors:** Paola Monti, Silvia Ravera, Andrea Speciale, Irena Velkova, Giorgia Foggetti, Paolo Degan, Gilberto Fronza, Paola Menichini

**Affiliations:** ^1^ Mutagenesis and Cancer Prevention Unit, IRCCS Ospedale Policlinico San Martino, Genoa, Italy; ^2^ Department of Experimental Medicine, University of Genoa, Genoa, Italy

**Keywords:** p53, energy metabolism, K120R mutation, antioxidant response, lipid peroxidation

## Abstract

The *TP53* tumor suppressor gene is one of the most studied gene in virtue of its ability to prevent cancer development by regulating apoptosis, cell cycle arrest, DNA repair, autophagy and senescence. Furthermore, the modulation of metabolism by P53 is fundamental for tumor suppressor activity. Studies in mouse models showed that mice carrying *TP53* mutations affecting the acetylation in the DNA binding domain still retain the ability to transactivate genes involved in metabolism. Noteworthy, mice expressing the triple 3KR or the single K117R mutant do not show early on-set tumor development in contrast to *TP53* ^*−/−*^ mice. Interestingly, the mouse K117R mutation corresponds to the human tumor-derived K120R modification, which abrogates P53-dependent activation of apoptosis without affecting growth arrest. In this study, we investigated the property of the human P53 K120R mutant in the regulation of metabolism by analyzing the transcriptional specificity in yeast- and mammalian-based reporter assays, the metabolic phenotype associated to its expression in colon cancer HCT116^*TP53*−/−^ cells and the induction of P53 targets and proteins involved in the antioxidant response. These properties were analyzed in comparison to wild type P53 protein, the human triple mutant corresponding to mouse 3KR and the cancer hot-spot R273H mutant. We confirm the selective functionality of P53 K120R mutant, which shows a transcriptional activity on cell cycle arrest but not on apoptotic targets. Interestingly, this mutant shows a partial transactivation activity on p53 response element belonging to the metabolic target TIGAR. Moreover, we observe a significant uncoupling between oxygen consumption and ATP production associated with higher lipid peroxidation level in all P53 mutants carrying cells with respect to wild type P53 expressing cells. Noteworthy, in the absence of a pro-oxidative challenge, cells expressing K120R mutant retain a partial capacity to modulate glucose metabolism, limiting lipid peroxidation with respect to the other P53 mutants carrying cells. Lastly, especially in presence of human 3KR mutant, a high expression of proteins involved in the antioxidant response is found. However, this response does not avoid the increased lipid peroxidation, confirming that only wild type P53 is able to completely counteract the oxidative stress and relative damages.

## Introduction

The *TP53* tumor suppressor gene, encoding the P53 tetrameric transcription factor, is the most commonly altered gene in human cancer, highlighting its key role in maintaining cellular homeostasis. The majority of *TP53* alterations found in tumors are missense mutations affecting six codons located in the central DNA binding domain (DBD) (i.e., 175, 245, 248, 249, 273, 282 referred as hot spots) that typically result in the complete loss of tumor suppressor functions. However, the spectrum of missense mutations is extremely broad along the *TP53* coding sequence, with more than 2,000 different amino-acid changes reported; indeed, *TP53* mutations can be classified not only as loss of function (LoF) but also as partial function, with altered specificity or gain of function (GoF), highlighting the functional heterogeneity of the encoded P53 mutant proteins (Leroy et al., 2013; Bisio et al., 2014; Monti et al., 2020b).

The *TP53* missense mutation, giving rise to the Lysine (K) to Arginine (R) substitution at codon 120 (here indicated as p53^K120R^), is an example of a DNA contact alteration found in cancers able to maintain some DNA binding capacity in virtue of the positive charge preservation of the amino acid side chain, which allows a heterogenous transcriptional activity (Liu et al., 2019; Xia et al., 2022). Indeed, it has been reported that the p53^K120R^ mutant is functionally active on cell cycle arrest target genes but not on apoptotic ones. This amino acid substitution, avoiding the acetylation of the K120 residue, results in the inability of the mutant protein to induce apoptosis (Sykes et al., 2006; Tang et al., 2006). Recently, a GoF activity for p53^K120R^ was also described; in fact, p53^K120R^ but not wild type P53 (p53^WT^) is able to bind and activate the transcription of the pro-survival *TNFAIP8* gene, that is critical for escape from apoptosis in tumor cells (Monteith et al., 2016).

For a long time, apoptosis, cell cycle arrest, and senescence were believed to mediate the tumor suppressive functions of P53; recently, it was highlighted that other pathways play a role in P53 ability to function as a tumor suppressor. Among these, the involvement of p53^WT^ in the aerobic energy metabolism modulation emerged, due to its ability to inhibit glucose transport, glycolysis, and fatty acid synthesis, but to promote lipid uptake, fatty acid oxidation, oxidative phosphorylation, and glutaminolysis (Lacroix et al., 2020; Liu and Gu, 2021). Furthermore, P53 has a crucial role in regulating ferroptosis, an iron-dependent form of non-apoptotic cell death (Liu and Gu, 2022; Xia et al., 2022).

Studies in mouse models showed that mice carrying K to R mutations at three acetylation sites in the central DBD of P53 (K117R; K161R; K162R encoding the so called p53^3KR^) and comprising the K117 site that corresponds to K120 codon of human P53, have impaired P53 function in apoptosis, cell cycle arrest, and senescence. However, these mice are not prone to early tumor development as the *TP53* null mice (Li et al., 2012; Xia et al., 2022). Interestingly, the p53^3KR^ mutant retains the ability as the p53^WT^ protein to transactivate some P53 target genes involved in the regulation of metabolism, including *TIGAR* and *GLS2*, coding for proteins that play critical role in glycolysis and mitochondrial respiration (Li et al., 2012; Xia et al., 2022). In addition, the cystine/glutamate antiporter *SLC7A11* (named also *xCT*) was identified from microarray analysis as a P53 target repressed by mouse p53^3KR^ mutant and p53^WT^ (Jiang et al., 2015). Interestingly, *xCT* has been found to be involved in ferroptosis, where lipid peroxidation plays a central role (Yang and Stockwell, 2016; Xie et al., 2017; Koppula et al., 2021; Liu and Gu 2022; Xia et al., 2022).

Conversely, defective P53-mediated apoptosis but efficient cell cycle-arrest and senescence were observed in mouse carrying the K117R mutation (here indicated as p53^K117R^), according to the inability of this mutant protein to induce the apoptotic targets *BBC3* (*PUMA*), *TNFRSF10B* (*KILLER*) and *PMAIP1* (*NOXA*) and the ability to induce the cell-cycle inhibitor *CDKN1A* (*P21*). As for p53^WT^ mice, p53^K117R^ animals did not develop early-onset tumors (Li et al., 2012; Liu et al., 2019; Liu and Gu 2022; Xia et al., 2022).

Although the functional specificity of the p53^K120R^ protein described above has been extensively studied, its contribution to metabolism modulation remains to be explored. Based on this premises, we investigated the property of human p53^K120R^ mutant, corresponding to mouse p53^K117R^, in the regulation of metabolism by analyzing i) its transcriptional specificity in yeast- and mammalian-based reporter assays, ii) the metabolic phenotypes of human colon cancer HCT116^*TP53*−/−^ cells expressing the mutant protein, and iii) the induction of P53 targets and proteins involved in the antioxidant response. The p53^K120R^ features were analyzed in comparison not only to p53^WT^ but also to the human P53 mutant resembling the triple mutant previously analyzed in mouse models. Furthermore, since the mutation giving rise to R273H DNA contact mutant protein (here indicated as p53^R273H^) is one of the main hot spot mutation found in human tumors, generating a mutant P53 protein unable to transactivate all type of P53 response elements (REs) or promoters (Loss of function, LoF) (Monti et al., 2020b), the metabolic functionality of p53^R273H^ was also studied.

## Materials and methods

### Yeast strains, expression vectors and reporter assay

The following yeast *S. cerevisiae* LUC1 reporter strains were used: yLFM-P21-5’, yLFM-MDM2P2C, yLFM-BAX A + B, yLFM-KILLER, yLFM-AIP1, yLFM-NOXA, and yLFM-TIGAR (Inga et al., 2002; Resnick and Inga, 2003). The strains are isogenic with the exception of the p53 Response Element (RE), a 20–22 bp nucleotide sequence that drives the expression of the firefly reporter gene (*LUC1*); the REs belong to apoptotic [*BAX*, *WDR1* (*AIP1*), *NOXA* and *KILLER*], cell cycle arrest [*CDKN1A* (*P21*)], P53 regulation (*MDM2*), and metabolic (*TIGAR*) targets. Yeast plasmids expressing the different *TP53* mutations were constructed exploiting *in vivo* yeast homologous recombination and all manipulations involving the reporter assay and yeast transformations were performed as previously described (Marengo et al., 2018; Monti et al., 2020a, 2019).

A pTSG-based vector was used in order to express P53 proteins (p53^WT^, p53^K120R^, p53^3KR^, p53^R273H^) under a galactose inducible expression (8 h, 0.128% Galactose); pRS314 was used as empty vector. Regarding the construction of the human P53 mutant that resembles the mouse p53^3KR^ mutant, since the mouse position K162 is replaced in human by Q165, an acetyl-mimetic amino acid, we constructed the human triple P53 K120R + K164R + Q165R mutant and named it p53^3KR^ for the analogy with the mouse mutant (Supplementary Figure S1).

### Mammalian cells line, expression vectors and reporter assay

Human colon carcinoma HCT116^*TP53*−/−^ cells were obtained from Dr. B. Vogelstein (The Johns Hopkins Kimmel Cancer Center, Baltimore, MD). Cells were grown in RPMI containing 10% foetal bovine serum, l-glutamine, penicillin-streptomycin antibiotic mixture (Euroclone, Milano, Italy), and maintained at 37 °C, in 5% CO2, at 100% humidity.

A pCIneo-based vector (Promega) was used to express P53 proteins (p53^WT^, p53^K120R^, p53^3KR^, p53^R273H^) in mammalian cells. Plasmids expressing P53 mutants were constructed as previously described (Marengo et al., 2018; Monti et al., 2020a). The pGL3-P21, pGL3-MDM2 and pGL3-BAX were available as reporter vectors; the pRL-SV40 plasmid was used as normalization vector. For reporter assays, HCT116^*TP53*−/−^ cells were transfected in medium free serum with 200 ng of pCIneo-based P53 expression vector, 250 ng of reporter vector, 50 ng of normalization vector in presence of Mirus Bio™ TransIT™-LT1 Transfection Reagent (Reagent:DNA ratio 3:1). After 24 h, cells were collected and washed with cold phosphate buffer saline (PBS). Lysis was performed in 1X PLB buffer (Passive Lysis Buffer, Promega). Luciferase assays were conducted, as previously described (Marengo et al., 2018; Monti et al., 2020a). For all reporter assay (yeast- and mammalian-based) wild type and mutant P53 fold induction over empty vector (pRS314 or pCIneo, respectively) were used to calculate the transactivation ability (as percentage, %) of the P53 mutant protein with respect to p53^WT^ (100%). For western blot and metabolic analysis, HCT116^*TP53*−/−^ cells were transfected as reported above with 1 µg of pCIneo-based P53 expression vector. After 16 h of transfection, cells were treated with 100 µM hydrogen peroxide (H_2_O_2_) for further 6 h. The percentage of living cells following this treatment did not differ among cells carrying different P53 proteins (Supplementary Figure S2). The use of pTSG- and pCINeo-based vectors for all experiments guarantee an equal expression of wild type and mutant P53 proteins (3).

### Western blotting analysis

Mammalian protein extracts were prepared as previously described (Marengo et al., 2018; Foggetti et al., 2019). 20–50 μg of mammalian cell lysates were resolved on 4–15% Mini protean TGX precast gels and transferred to nitrocellulose membranes by Trans-Blot Turbo Blotting System (Bio-Rad, Milano, Italy). Membranes were blocked with 2% non-fat dry milk in 0.1% Tween-20 in PBS for 1 h, then incubated at room temperature (1 h) or overnight at 4 °C with the appropriate primary antibody. The following antibodies were employed: anti-P53 (clone DO-1, Santa Cruz Biotechnology), anti-MDM2 (clone SPM14, sc-965 Santa Cruz), anti-P21 Waf1/Cip1 (DCS60 #2946 Cell Signaling Technology), anti-BAX (clone N-20, sc-493 Santa Cruz), anti-TIGAR (clone E-2, sc-166290 Santa Cruz), anti-G6PD (D5D2, #12263 Cell Signaling Technology), anti-GCLM (ab154017, Abcam), anti-GCLC (clone EP13475, ab190685 Abcam), anti-NRF2 (clone 21HCLC, #710574 Invitrogen), anti-xCT/SLC7A11 (Clone D2M7A, #12691 Cells Signaling Technology), anti-human β-Actin (clone AC-74, Sigma–Aldrich). The appropriate IgG-horseradish peroxidase-conjugated secondary antibodies (anti-mouse or anti-rabbit IgG HRP, A9044 and A9169 respectively, Sigma-Aldrich) were used. Detection was carried out with ECL FAST PICO (ECL-1002, Immunological Sciences, Roma, Italy). Chemiluminescence was analyzed by Alliance LD, UVITEC Cambridge (Cambridge, U.K.).

### Oxygen consumption rate and ATP synthesis evaluation

Oxygen consumption rate (OCR) was evaluated in 100,000 cells for each experiment by a thermostatically controlled oxygraph apparatus equipped with an amperometric electrode (Unisense-Microrespiration, Unisense A/S). Cells were resuspended in PBS and permeabilized with 0.03% digitonin. To evaluate the OCR performed by Complexes I, III and IV pathway, 10 mM pyruvate +5 mM malate were added, while 20 mM succinate were used to activate the pathways composed by Complexes II, III and IV; after the addition of respiratory substrates, 0.1 mM ADP was added in each experiment. The respiratory rates were expressed as nmol O/min/10^6^ cells (Cappelli et al., 2020).

To evaluate the mitochondrial ATP synthesis, the F_o_-F_1_ ATP synthase activity was measured in 100,000 cells incubated for 10 min at 37°C in a medium containing 10 mM Tris-HCl pH 7.4, 100 mM KCl, 5 mM KH_2_PO_4_, 1 mM EGTA, 2.5 mM EDTA, and 5 mM MgCl_2_, 0.6 mM ouabain, 0.3 mM P1, P5-Di (adenosine-5′) pentaphosphate, and 25 mg/ml ampicillin. The ATP synthesis was induced by the addition of 10 mM pyruvate +5 mM malate or 20 mM succinate, to stimulate the pathways composed by Complexes I, III and IV or Complexes II, III and IV, respectively. The reaction was monitored for 2 min, every 30 s, in a luminometer (GloMax^®^ 20/20n Luminometer, Promega Italia), by the luciferin/luciferase chemiluminescent method (luciferin/luciferase ATP bioluminescence assay kit CLSII, Roche), with ATP standard solutions between 10^−8^ and 10^−5^ M. Data were expressed as nmol ATP produced/min/10^6^ cells (Cappelli et al., 2020). To verify that OCR and ATP synthesis are dependent on OxPhos machinery, samples were treated with 1 μM Rotenone or 10 μM Antimycin A, specific inhibitors of Complex I and Complex III, respectively (Supplementary Figure S4).

By measuring the OCR and the ATP synthesis trough F_o_-F_1_ ATP synthase, the oxidative phosphorylation (OxPhos) efficiency was calculated as the ratio between the produced ATP concentration and the consumed oxygen amount (P/O ratio).

### Glucose consumption, lactate release assay, and lactate dehydrogenase activity

To evaluate the glucose consumption, the glucose content in the growth medium was evaluated following the reduction of NADP at 340 nm, by the hexokinase (HK) and glucose six phosphate dehydrogenase (G6PD) coupling system. The assay medium contained 100 mM Tris-HCl pH 7.4, 2 mM ATP, 10 mM NADP, 2 mM MgCl2, 2 International Units (IU) of hexokinase, and 2 IU of glucose 6-phosphate dehydrogenase. Data were normalized on the cell number and expressed as mM consumed glucose/10^6^ cells (Cappelli et al., 2017).

Lactate concentration was assayed spectrophotometrically in the growth medium, following the reduction of NAD+, at 340 nm. The assay medium contained 100 mM Tris-HCl pH 7.4, 5 mM NAD+, and 1 IU/ml of lactate dehydrogenase (LDH). Data were normalized on the cell number and expressed as mM released lactate/10^6^ cells (Cappelli et al., 2017).

Once the glucose consumption and lactate release amounts have been obtained, the lactate fermentation yield was calculated by comparing the values of lactate produced with those of theoretical lactate calculated as twice the glucose consumed.

Lactate dehydrogenase activity was evaluated as a marker of lactate fermentation, following the NADH oxidation, at 340 nm. The reaction mixtures contained: 100 mM Tris-HCl pH 7.4, 0.2 mM NADH, and 5 mM pyruvate (Cappelli et al., 2020). For the assay 20 µg of cell homogenate, obtained by sonication of cells resuspended in PBS, were employed.

### Evaluation of malondialdehyde content

Malondialdehyde (MDA) concentration was assayed as a lipid peroxidation marker, by the thiobarbituric acid reactive substances (TBARS) assay (Colla et al., 2016). 600 μl of TBARS solution was added to 50 μg of cell homogenate dissolved in 300 μl of Milli-Q water. The mix was incubated for 40 min at 100 °C. Afterwards, the sample was centrifuged at 14,000 rpm for 2 min, and the supernatant was analyzed spectrophotometrically at 532 nm.

### Statistical analysis

T student test, ordinary One-way anova and Two-way anova followed by comparisons test were performed by using GraphPad Prism version 8.0. For Windows, GraphPad Software, San Diego, California, United States .

## Results

### The human p53^K120R^, p53^3KR^, and p53^R273H^ mutants show different transactivation activity towards P53 targets

**FIGURE 1:**
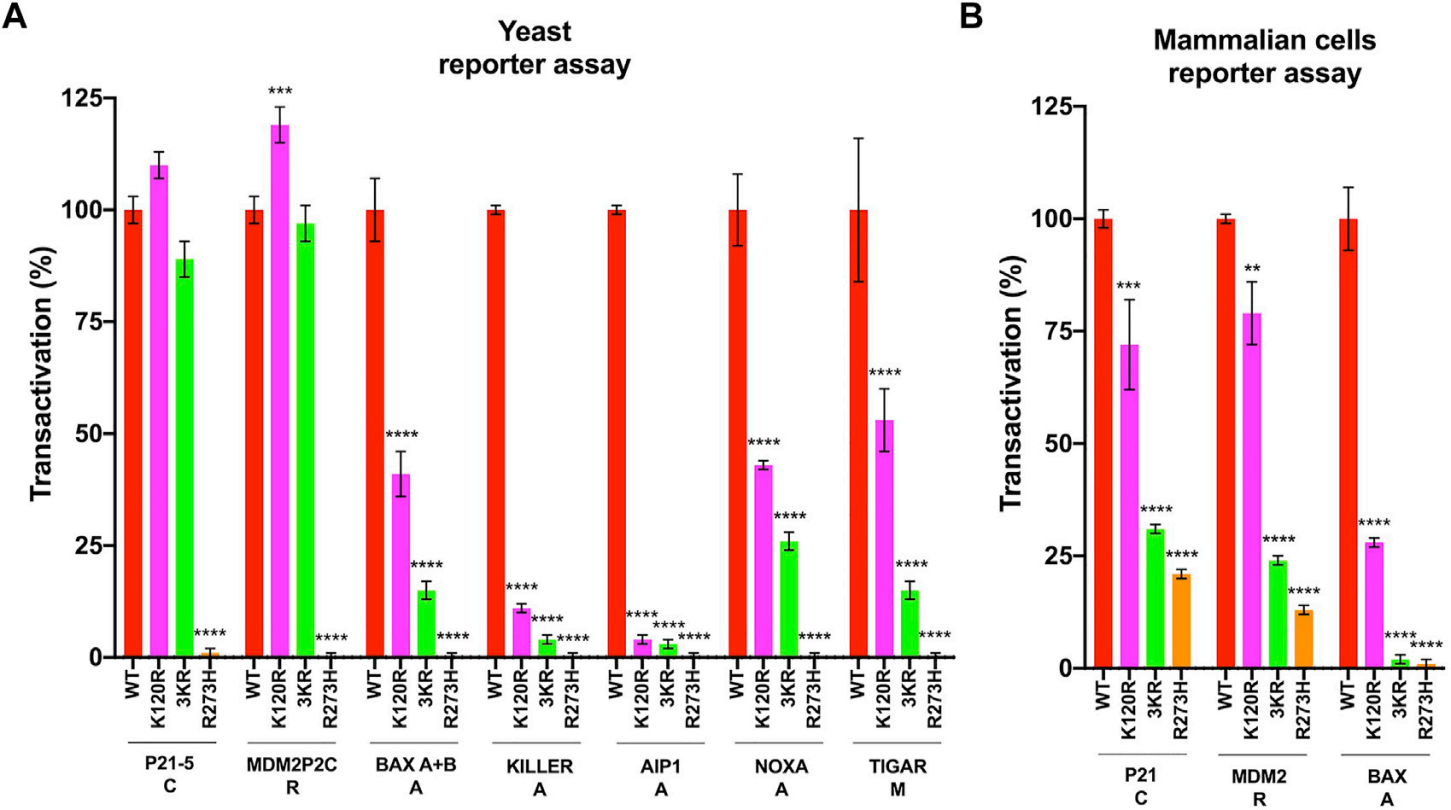
Transactivation activity of p53^WT^, p53^3KR^, p53^K120R^, and p53^R273H^ proteins. **(A)** p53^K120R^, p53^3KR^, p53^R273H^ mutant proteins transactivation ability towards seven p53 REs with respect to p53^WT^ in yLFM yeast strains. **(B)** p53^K120R^, p53^3KR^, p53^R273H^ mutant proteins transactivation ability towards three p53 responsive promoters with respect to p53^WT^ in HCT116^*TP53*−/−^ cell line. Data of reporter assays are presented as mean ± standard deviation (SD) of two independent experiments with three biological replicates. The symbols **, *** and **** indicate significant differences for *p* = 0.0015, *p* ≤ 0.001 and *p* < 0.0001, respectively, between cells expressing p53^WT^ (WT) and cells expressing p53^K120R^ (K120R), p53^3KR^ (3KR) or p53^R273H^ (R273H). Functional classification of the gene associated with the p53 REs or promoters is indicated as following: C, cell cycle arrest; R, P53 regulation; A, apoptosis; M, metabolism.

The functional activity of the human p53^K120R^, p53^3KR^ and p53^R273H^ mutants was examined using a yeast-based functional assay. Our analyses confirm that p53^K120R^ maintains high functionality on *P21* and *MDM2* REs but shows partial (*BAX* and *KILLER*) or poor (*AIP1*, *NOXA*) activity on the REs of apoptotic genes (Figure 1A). The human p53^3KR^ mutant shows a similar or a lower functionality (p53^K120R^ > p53^3KR^) on the same REs. The p53^R273H^ mutant, used as a control of LoF mutant, shows no transactivation activity in any yeast strain tested. The p53^K120R^ and p53^3KR^ mutants were also evaluated on the RE of the metabolic target *TIGAR*; they show a partial activity (p53^K120R^ > p53^3KR^) and not a complete loss of transactivation ability as displayed by p53^R273H^ mutant (Figure 1A).

The transactivation specificity toward *P21*, *MDM2*, and *BAX* targets was confirmed with a mammalian cells-based reporter assay in colon cancer HCT116^*TP53*−/−^ cells expressing mutant P53 proteins (Figure 1B). The p53^K120R^ is able to transactivate the *P21* and *MDM2* promoters but less efficiently the *BAX* promoter. On the contrary, the p53^3KR^ mutant loses 70–80% of its activity on all promoters, showing a behavior more similar to p53^R273H^ mutant (Figure 1B).

**FIGURE 2:**
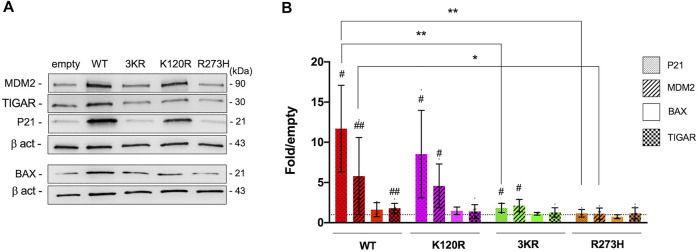
Modulation of endogenous P53 targets by p53^WT^, p53^K120R^, p53^3KR^, and p53^R273H^ in HCT116^*TP53*−/−^ cells. **(A)** Representative western blots showing the level of MDM2, TIGAR, P21, BAX and Beta-actin (β-act) endogenous proteins in HCT116^*TP53*−/−^ cells transiently expressing p53^WT^, p53^3KR^, p53^K120R^, and p53^R273H^. **(B)** Histogram represents the amount of P21, MDM2, BAX, and TIGAR proteins detected in HCT116^*TP53*−/−^ transfected cells and normalized for β-act. Data are reported as mean ± SD and are obtained from at least three independent experiments. The levels of the different proteins are calculated as fold over the level of the same proteins found in cells transfected with the empty vector (#*p* < 0.05; ##*p* < 0.005; t Student); the dotted line corresponds to one-fold over empty. The symbols * and ** indicate significant differences of protein expression (with *p* = 0.0130 and *p* ≤ 0.0091, respectively) between cells expressing p53^WT^ (WT) and cells expressing p53^K120R^ (K120R), p53^3KR^ (3KR) or p53^R273H^ (R273H). MDM2 induction by p53^3KR^ results not quite significantly different (*p* = 0.0563) from the corresponding induction by p53^WT^.

In the same mammalian cells, the level of P21, MDM2, BAX, and TIGAR endogenous proteins was also assessed (Figure 2). In keeping with the functional assays described above, both p53^WT^ and p53^K120R^ efficiently induce the endogenous P21 and MDM2 proteins (Figures 2A,B), whereas p53^3KR^ retains low ability with a behavior resembling the p53^R273H^ mutant. The induction of BAX is not significant in any conditions. Regarding TIGAR, the induction is significant only in cells expressing p53^WT^ (1.8-fold over empty), but not in cells expressing the other P53 mutant proteins.

All together these data confirm the selective functionality of p53^K120R^ mutant protein with respect to p53^3KR^ and p53^R273H^ mutants.

### The human p53^K120R^, p53^3KR^, and p53^R273H^ mutants determine an altered metabolic phenotype

To evaluate the metabolic phenotype induced by the expression of P53 mutant proteins (p53^K120R^, p53^3KR^, and p53^R273H^), the OxPhos activity and the lactate fermentation rate were investigated in HCT116^*TP53*−/−^ cells transiently transfected as previously described.

**FIGURE 3:**
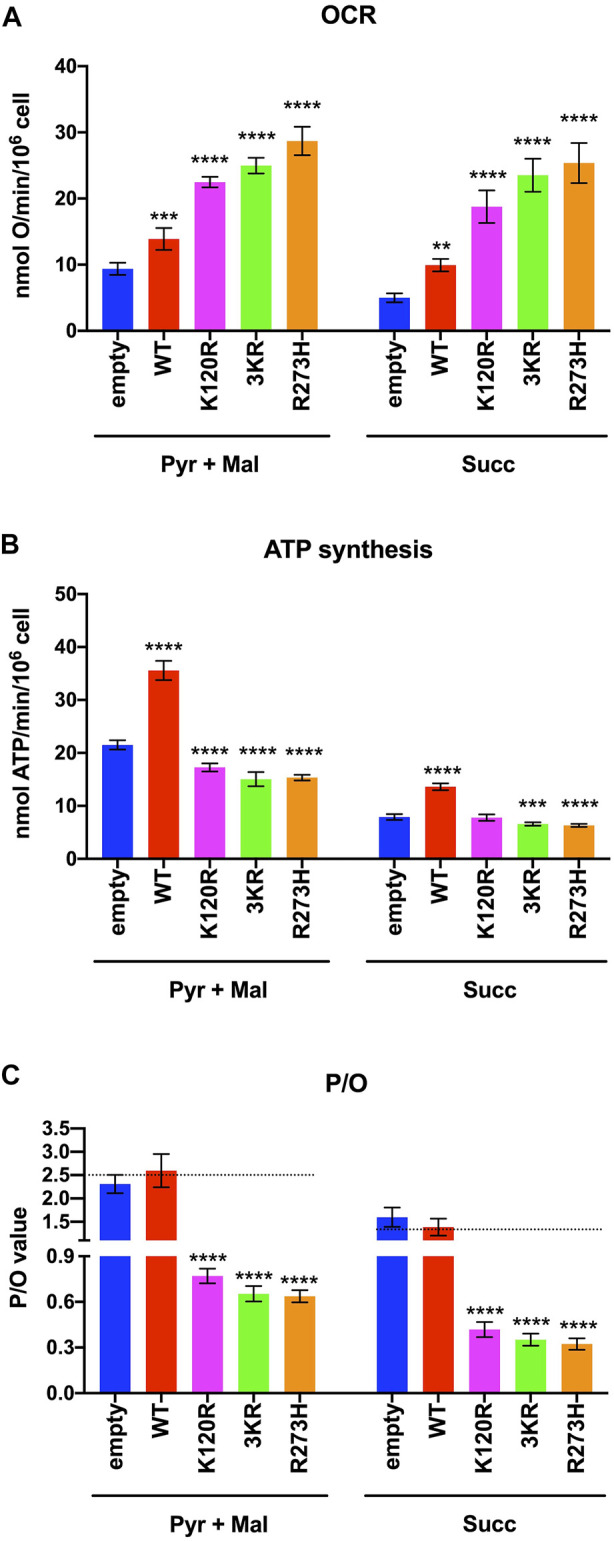
OxPhos function in HCT116^*TP53*−/−^ cells expressing p53^WT^, p53^3KR^, p53^K120R^, and p53^R273H^ proteins. **(A)** OCR in the presence of Pyruvate (Pyr) plus Malate (Mal) or Succinate (Succ). **(B)** ATP synthesis through the F_o_-F_1_ ATP synthase in the same condition as above. **(C)** OxPhos efficiency as P/O ratio in the same condition as above. The dotted lines indicate the expected P/O ratio levels based on the employed respiratory substrates. Each panel is representative of at least four independent experiments and data are reported as mean ± SD. The symbols **, *** and **** indicate significant differences for *p* = 0.0048, *p* ≤ 0.0009 and *p* < 0.0001, respectively, between the control (empty) cells and those expressing p53^WT^ (WT), p53^K120R^ (K120R), p53^3KR^ (3KR) or p53^R273H^ (R273H), for each OxPhos stimulation condition (addition of pyruvate + malate or succinate).

The OxPhos function was assayed in terms of OCR and ATP synthesis, in the presence of the respiratory substrate pyruvate plus malate or succinate. As reported in Figure 3A, the OCR appears increasingly and significantly higher in p53^WT^, p53^K120R^, p53^3KR^, and p53^R273H^ expressing cells compared to the cells transfected with the empty vector. Moreover, the expression of all type of P53 mutants causes at least a two-fold increase in OCR than the one measured in p53^WT^ carrying cells. Conversely, the ATP synthase activity significantly increases only in cells expressing the p53^WT^, while in the presence of all P53 mutant proteins it is slightly lower or comparable to cells transfected with empty vector (Figure 3B).

The evaluation of the P/O ratio as a marker of OxPhos efficiency (Figure 3C) shows that only empty vector and p53^WT^ expressing cells have values similar to those reported when the oxygen consumption is entirely devoted to energy production (e.g., 2.5 with pyruvate plus malate or 1.5 with succinate, Hinkle, 2005). Conversely, cells expressing p53^K120R^, p53^3KR^, and p53^R273H^ display lower P/O values, suggesting the uncoupling between oxygen consumption and ATP production.

The impairment of the mitochondrial energy functions can lead to the anaerobic glycolysis increase, as an attempt to maintain a stable energy status and to recycle the reduced coenzymes (e.g., NADH), converting pyruvate to lactic acid by LDH activity (Prochownik and Wang, 2021; Ravera et al., 2021). Thus, the lactic acid fermentation extent was determined in the same cells and conditions by measuring the glucose consumption and the released lactate amount in the culture medium (i.e., lactic acid in solution), as well as the LDH activity.

**FIGURE 4:**
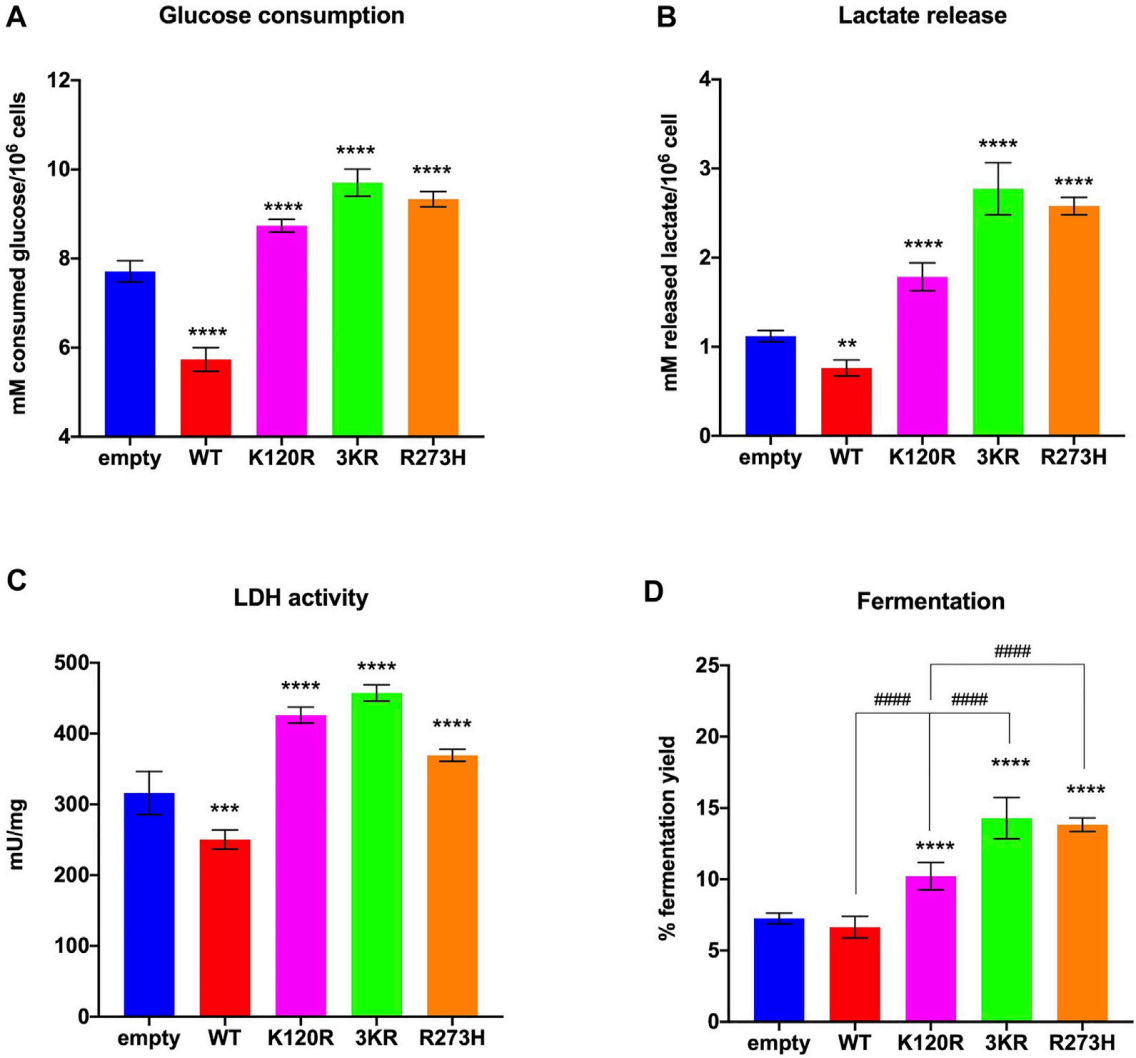
Anaerobic metabolism in HCT116^*TP53*−/−^ cells expressing p53^WT^, p53^3KR^, p53^K120R^, and p53^R273H^ proteins. **(A)** Glucose consumption. **(B)** Lactate release in the medium. **(C)** LDH activity. **(D)** Percentage of lactic acid fermentation yield. Each panel is representative of at least four independent experiments; data are reported as mean ± SD. The symbols **, ***, **** indicate significant differences for *p* < 0.01, 0.001, and 0.0001, respectively, between the control (empty) cells and those expressing p53^WT^ (WT), p53^K120R^ (K120R), p53^3KR^ (3KR) or p53^R273H^ (R273H); the symbol #### indicates significant differences for *p* < 0.0001 between p53^K120R^ and the indicated samples.

In the presence of p53^WT^ glucose consumption, lactate release, and LDH activity are significantly lower than in cells transfected with the empty vector, while cells expressing p53^K120R^, p53^3KR^, and p53^R273H^ proteins show a significant increment (Figures 4A–C). These effects ultimately lead to a lower lactate fermentation yield in the presence of p53^WT^ (that is comparable to empty cells) with respect to P53 mutant proteins (Figure 4D) due to the corresponding enhancement of aerobic mitochondrial energy metabolism, as previously reported (Figure 3). Conversely, the increase in fermentation yield observed in the presence of the p53^K120R^, p53^3KR^, and p53^R273H^ mutant proteins indicates a metabolic switch from aerobic to anaerobic metabolism. Nevertheless, the observation that the p53^K120R^ mutant triggers a significant lower fermentation yield with respect to p53^3KR^ and p53^R273H^, although significantly higher than p53^WT^ (Figure 4D), may suggest that this mutant still preserves some capacity to modulate glucose metabolism.

We also determined the level of glutaminase (GLS), glutamate dehydrogenase activity (GDH), as well as 3-hydroxyacyl-CoA dehydrogenase as markers of amino acid metabolism and beta-oxidation, respectively. Data reported in Supplementary Figure S5 show that the activities of the three analyzed enzymes are significantly increased in p53^WT^ expressing cells compared to the control, confirming the central role of P53 in the modulation of amino acid and lipid metabolisms related to OxPhos (Simabuco et al., 2018). Conversely, cells expressing P53 mutant proteins (p53^K120R^, p53^3KR^, and p53^R273H^) are characterized by a lower activation of these two metabolic pathways with respect to p53^WT^, despite maintaining a higher activity than the empty control. In detail, p53^3KR^ displays lower enzymatic activities compared to p53^K120R^ carrying cells, which, in turn, appear slightly but significantly lower than those observed in p53^WT^ cells. These results are in line with the decreasing efficiency of aerobic metabolism and the need to increase glucose consumption and anaerobic glycolysis in cells expressing these P53 mutant proteins since both the conversion of glutamine to alpha-ketoglutarate and beta-oxidation are two metabolisms that provide substrates for the Krebs cycle.

**FIGURE 5:**
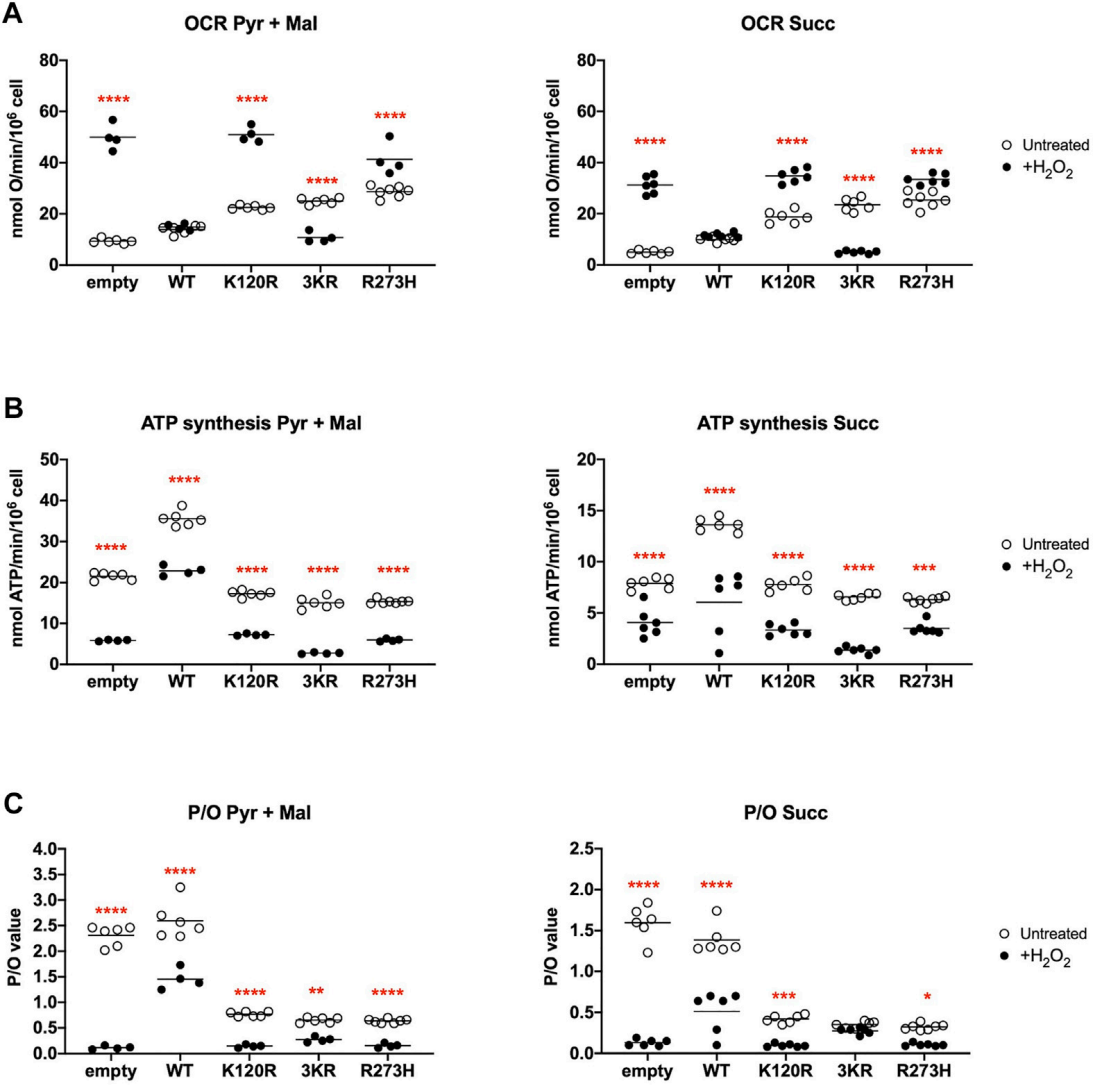
Comparison of OxPhos function in H_2_O_2_ treated HCT116^*TP53*−/−^ cells with untreated cells, expressing p53^WT^, p53^3KR^, p53^K120R^, and p53^R273H^ proteins. **(A)** OCR in untreated (empty dots) and 100 μM H_2_O_2_ treated (black dots) cells in the presence of Pyruvate (Pyr) plus Malate (Mal) or Succinate (Succ). **(B)** ATP synthesis through the F_o_-F_1_ ATP synthase in the same condition as above. **(C)** OxPhos efficiency as P/O ratio in the same condition as above. Each panel is representative of at least four independent experiments and data are reported as mean. The red symbols *, **, *** and **** indicate significant differences for *p* = 0.0110, *p* = 0.0026, *p* ≤ 0.0003 and *p* < 0.0001, respectively, between the untreated and 100 μM H_2_O_2_ treated samples. In each panel, the empty dots represent the data from untreated samples depicted in Figure 3.

To evaluate the energy metabolism modulation during an oxidative insult, we used hydrogen peroxide (H_2_O_2_) as pro-oxidative stress. The addition of H_2_O_2_ determines an increase of OCR values independently from the substrate in empty vector, p53^K120R^, and p53^R273H^ expressing cells compared to untreated cells but a decrease in cells with p53^3KR^ mutant (Figure 5A); only p53^WT^ expressing cells show a stable OCR. However, the described modulation of the oxygen consumption does not correspond to the ATP synthase activity, that shows a general decrease in all samples (Figure 5B). However, these last results highlight again the highest capacity of p53^WT^ expressing cells to synthesize ATP, even under oxidative stress conditions. Consequently, a general decrement of OxPhos efficiency in H_2_O_2_ treated compared to untreated samples (Figure 5C) is evident as demonstrated by the P/O values, being this decrease less significant for p53^3KR^ expressing cells; again, the presence of p53^WT^ ensures a higher OxPhos efficiency with respect to the comparable values of P/O from samples expressing P53 mutant proteins.

**FIGURE 6:**
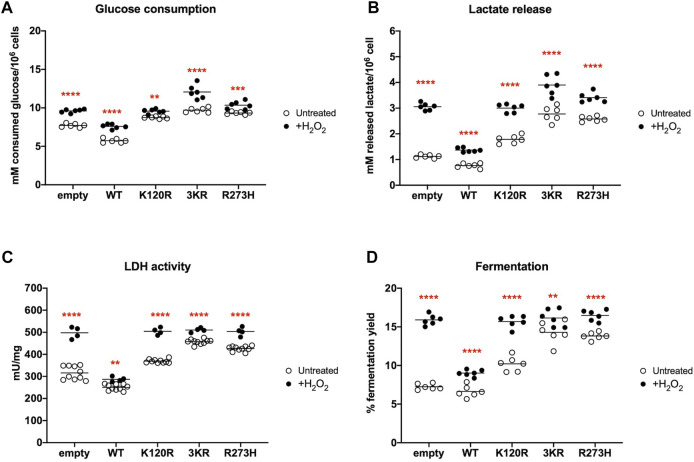
Comparison of anaerobic metabolism in H_2_O_2_ treated HCT116^*TP53*−/−^ cells with untreated cells expressing p53^WT^, p53^3KR^, p53^K120R^, and p53^R273H^ proteins. **(A)** Glucose consumption in untreated (empty dots) and 100 μM H_2_O_2_ treated (black dots) cells. **(B)** Lactate release in the medium in the same condition as above. **(C)** LDH activity in the same condition as above. **(D)** Percentage of lactic acid fermentation yield in the same condition as above. Each panel is representative of at least four independent experiments and data are reported as mean. The red symbols ** and **** indicate significant differences for *p* ≤ 0.0031 and *p* < 0.0001, respectively, between the untreated and 100 μM H_2_O_2_ treated samples. In each panel, the empty dots represent the data from untreated samples depicted in Figure 4.

This further uncoupling between the OCR and the ATP production leads to a general significant increase in glucose consumption, lactate release, LDH activity, and lactic acid fermentation yield in all H_2_O_2_ treated cells (Figures 6A–D). Notably, the p53^3KR^ cells seem to have almost reached the maximum of fermentation capacity in untreated conditions (Figure 6D). Following H_2_O_2_ treatment the presence of all three types of P53 mutant proteins causes a higher fermentation yield than the presence of the p53^WT^ protein, indicating a comparable altered metabolic phenotype in the presence of a pro-oxidative stress (Figure 6D).

### The human p53^K120R^, p53^3KR^ and p53^R273H^ mutants cause an increment of oxidative stress

The aerobic metabolism is associated with the production of ROS, which increases in uncoupled conditions (Cadenas and Davies, 2000; Turrens, 2003; Ravera et al., 2021). We measured the induction of ROS and the level of GSH in untreated HCT116^*TP53−/−*^ cells transiently expressing p53^WT^, p53^3KR^, p53^K120R^ and p53^R273H^ (Supplementary Figure S6). As reported in Supplementary Figure S6A, cells expressing p53^WT^, p53^K120R^, p53^3KR^, and p53^R273H^ displayed an increased ROS production compared to control cells, more evident in presence of mutant proteins than wild type P53 protein. However, while the oxidative stress in cell expressing p53^WT^ is associated with the increment of efficient OxPhos activity (Figure 3), the ROS production in P53 mutant proteins expressing cells depends on the uncoupling between the OCR and the ATP synthesis. The intracellular level of GSH, a marker of oxidative stress scavenger, was higher compared to the empty cells only in presence of wild type P53 protein (Supplementary Figure S6B). Interestingly, the ROS/GSH ratio, a value that indicates the balance between oxidative stress and antioxidant defence, increased from the p53^K120R^ to p53^R273H^ cells, with the p53^K120R^ expressing cells having the lowest value (Supplementary Figure S6C). This indicate that among the P53 mutants, the p53^K120R^ possess the best antioxidant defence, although less efficient than the p53^WT^.

Subsequently, the MDA concentration, a marker of lipid peroxidation, was evaluated to test whether the metabolic changes associated with the expression of p53^K120R^, p53^3KR^, and p53^R273H^ mutant proteins cause an increase of cellular oxidative stress compared to the presence of p53^WT^.

**FIGURE 7:**
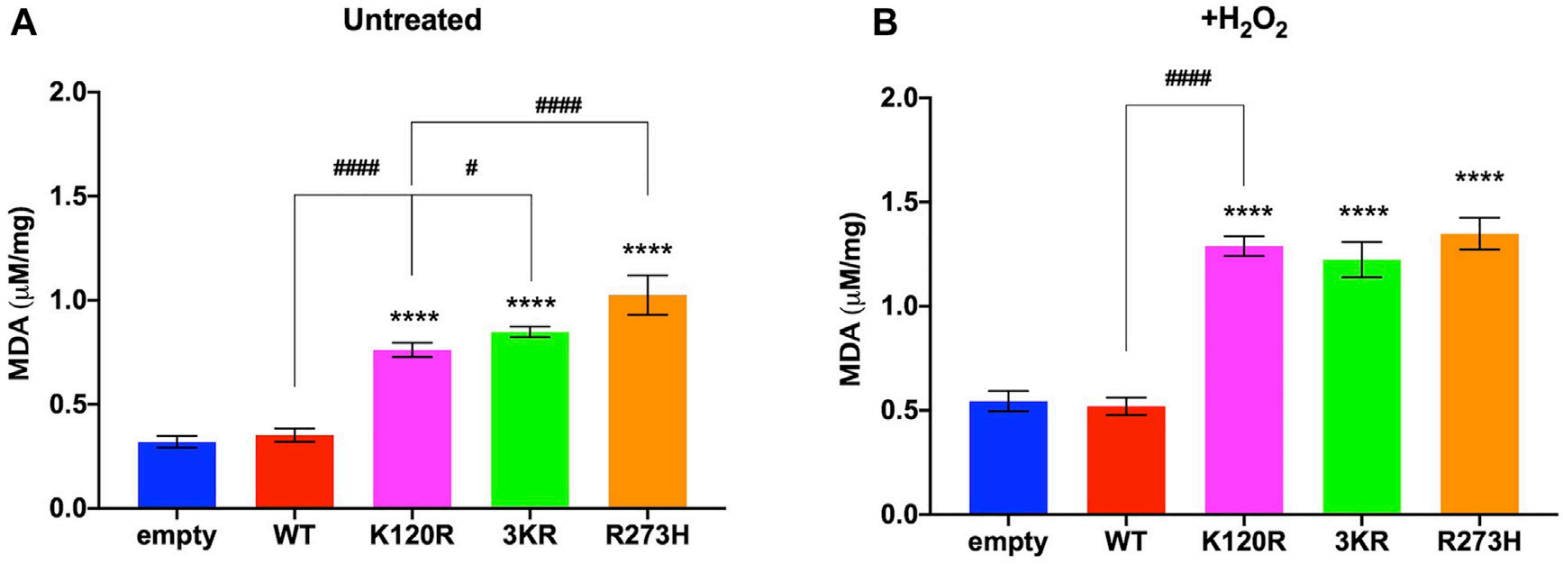
Evaluation of oxidative stress in untreated and H_2_O_2_ treated HCT^116*TP53*−/−^ cells expressing p53^WT^, p53^3KR^, p53^K120R^, and p53^R273H^ proteins. **(A)** MDA level in untreated cells. **(B)** MDA level in 100 μM H_2_O_2_ treated cells. The panels are representative of at least four independent experiments and data are reported as mean ± SD. The symbol **** indicates significant differences for *p* < 0.0001 between empty and the other samples; the symbols # and #### indicate significant differences for *p* = 0.0445 and *p* < 0.0001, respectively, between p53^K120R^ and the indicated samples.

In both untreated and H_2_O_2_ treated conditions, we observed an increase of MDA in P53 mutants but not in p53^WT^ expressing cells with respect to cells transfected with the empty vector (Figure 7A, B), although more pronounced following the pro-oxidative stress. This suggests that only the presence of p53^WT^ protein can counteract the oxidative stress to avoid basal and induced oxidative damages. Interestingly, in untreated conditions p53^K120R^ expressing cells show the lowest MDA accumulation among samples carrying mutant P53 proteins. These results are in keeping with the ROS/GSH ratio reported in Supplementary Figure S7 for untreated cells and confirm the lipid peroxidation as a reliable marker of the antioxidant defence capacity in this cell model.

**FIGURE 8:**
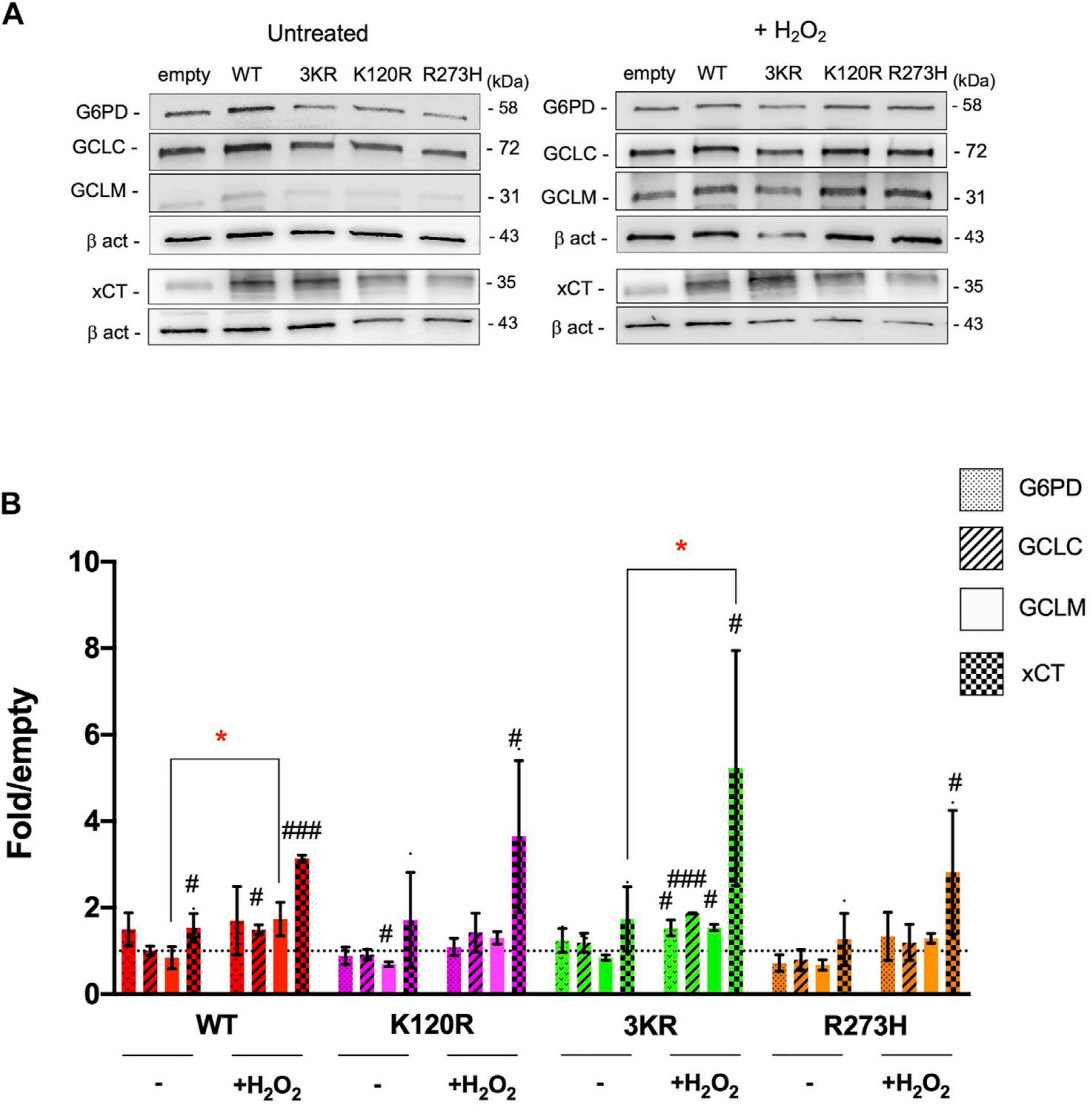
Modulation of proteins involved in the antioxidant response by p53^WT^, p53^3KR^, p53^K120R^, and p53^R273H^ proteins in untreated and H_2_O_2_ treated HCT116^*TP53*−/−^ cells. **(A)** Representative western blots showing the level of G6PD, GCLC, GCLM, xCT and Beta-actin (β-act) endogenous proteins in untreated and 100 μM H_2_O_2_ treated HCT116^*TP53*−/−^ cells transiently expressing p53^WT^, p53^K120R^, p53^3KR^, and p53^R273H^. **(B)** Histograms representing the amount of G6PD, GCLC, GCLM, and xCT proteins detected in HCT116^*TP53*−/−^ transfected cells in untreated or treated samples and normalized for β-act. Data were obtained after chemiluminescence analysis of western blots from at least two independent experiments and are reported as mean ± SD. The levels of the different proteins were calculated as fold over the level of the same proteins found in cells transfected with the empty vector (#*p* < 0.05; ##*p* < 0.005; ###*p* < 0.0005; t Student); the dotted line corresponds to one-fold over empty. The red symbol * indicates a significant difference for *p* ≤ 0.0295 between the H_2_O_2_ treated and the indicated untreated samples.

To evaluate whether cells carrying p53^K120R^, p53^3KR^, and p53^R273H^ mutant proteins activated the antioxidant response to counteract the increment of oxidative stress associated to the metabolic changes, the expression of G6PD and xCT proteins was assessed (Figure 8). Specifically, G6PD is the first enzyme of the pentose phosphate pathway, providing the NADPH (reduced nicotinamide adenine dinucleotide phosphate) necessary for the GSSG (glutathione disulfide) reduction, and xCT is one of the principal transporters for cystine, a key component of GSH (Clemons et al., 2017; Yang et al., 2019). Expression of G6PD and xCT has been identified under P53 modulation with different mechanisms (Jiang et al., 2015, 2011; Xie et al., 2017). In addition, although not direct targets of P53, the level of GCLM and GCLC were measured since they are important player in the synthesis of GSH (Lu, 2013).

In untreated cells, only a significant induction of xCT expression in p53^WT^ expressing cells is measured (1.5-fold). No other significant P53-dependent modulations are evident with exception of a downregulation of GCLM level in p53^K120R^ carrying cells with respect to empty sample (Figures 8A,B). Following H_2_O_2_ treatment, a significant increase in xCT expression is associated to every condition (p53^WT^, p53^K120R^, p53^3KR^ and p53^R273H^). Moreover, while p53^WT^ cells are characterized also by an upregulation of GCLC, p53^3KR^ cells show a significant induction of all analyzed proteins (Figures 8A,B). Lastly, when treated cells are compared to untreated, p53^WT^ and p53^3KR^ expressing cells show a significant increment of GCLM and xCT, respectively (Figures 8A,B).

The xCT increase is likely independent from change in level of NRF2 transcription factor, a known modulator of xCT, since its expression, although slightly increases following H_2_O_2_ treatment, does not significantly change in any transfection and treatment conditions (Supplementary Figure S7).

Lastly, the level of GPX4, known to be a marker of ferroptosis, was also measured to specifically measure a possible modulation of its level in P53^WT^, P53^K120R^, P53^3KR^ and P53^R273H^ expressing cells before and after treatment with H_2_O_2_. As reported in Supplementary Figure S8, in these treatment conditions the modulation of GPX4 was comparable in P53^K120R^ and P53^WT^ carrying cells. Slightly lower, although not significant, level of GPX4 was found in P53^3KR^ expressing cells.

All together these results could indicate that, especially in the p53^3KR^ expressing cells, an antioxidant response is activated with the tentative to balance the increment of oxidative stress. However, this response is not sufficient to avoid the lipid peroxidation as indicated by the previously observed MDA accumulation.

## Discussion

In this paper, we investigated the property of human p53^K120R^ mutant protein, corresponding to the p53^K117R^ in mouse, in the regulation of metabolism by analyzing its transcriptional ability in yeast and mammalian reporter assays, the metabolic phenotype in HCT116^*TP53*−/−^ cells expressing the mutant protein and the corresponding induction of P53 targets and proteins involved in the antioxidant response, before and after a pro-oxidative challenge (i.e., H_2_O_2_). In addition, the human P53 triple mutant (p53^3KR^), that resembles the mouse mutant carrying Lysine to Arginine missense mutations at the three acetylation sites of the DBD (Li et al., 2012; Liu et al., 2019; Xia et al., 2022), and the well-known LoF and GoF p53^R273H^ mutant, were studied. The properties of P53 mutants were compared to p53^WT^, whose contribution in metabolism regulation has been recently demonstrated (Lacroix et al., 2020; Liu and Gu, 2022, 2021)

In yeast and mammalian reporter assays p53^K120R^ protein is able to transactivate the *P21* and *MDM2* REs (yeast) or promoters (mammalian cells) but poorly or partially *BAX*, *KILLER*, *AIP1*, *NOXA* targets, confirming its transcriptional specificity (Figure 1); indeed, p53^K120R^ has been previously characterized for its ability to induce cell cycle arrest targets and for its deficiency in activation of apoptosis genes (Sykes et al., 2006; Tang et al., 2006). The p53^3KR^ mutant loses transactivation activity with respect to p53^K120R^, but shows a behavior more similar to the p53^R273H^ mutant in HCT116^*TP53*−/−^ cells, where it decreases its activity on all promoters. The results obtained in reporter assays are in keeping with the expression of the same endogenous P53 target proteins (P21, MDM2, BAX) in HCT116^*TP53*−/−^ cells transfected with p53^WT^, p53^K120R^, p53^3KR^ and p53^R273H^ proteins (Figure 2). Moreover, p53^K120R^ is not able to induce the endogenous *TIGAR*; however, since only a 1.8-fold increase of TIGAR protein is detected in HCT116 cells by p53^WT^ (Figure 2) and approximately a 50% of the wild type transcriptional activity is maintained by p53^K120R^ on *TIGAR* RE in the functional yeast assay (Figure 1), we might speculate that the induction of TIGAR protein in human cells expressing the p53^K120R^ mutant is undetectable.

The induction of TIGAR by p53^WT^ can contribute to reduce the rate of glycolysis and to regulate ROS at cellular level (Bensaad et al., 2006). Consequently, the partial ability of p53^K120R^ mutant to activate the *TIGAR* RE, as observed in the yeast functional assay, could have an impact on glucose metabolism measured in HCT116^*TP53*−/−^ transfected cells. Our results show that although all mutant P53 proteins carrying cells switch from aerobic to anaerobic metabolism (Figures 3, 4), the p53^K120R^ mutant triggers a lower percentage of fermentation with respect to p53^3KR^ and p53^R273H^. This suggests that p53^K120R^ still conserves some capacity to modulate glucose metabolism and limit the fermentation yield, although such peculiar features are lost following the treatment with H_2_O_2_, as highlighted by the similar behavior of all P53 mutant proteins in pro-oxidant conditions (Figures 5, 6).

MDA level is known as a marker of oxidative stress, which increases when mitochondria are in uncoupled conditions (Cadenas and Davies, 2000; Turrens, 2003; Ravera et al., 2021). Interestingly, p53^K120R^ expressing cells show the lowest MDA accumulation within samples carrying P53 mutant proteins in untreated conditions (Figure 7), suggesting again a partial capacity by p53^K120R^ to counteract the oxidative stress to avoid oxidative damages. As expected, p53^WT^ cells are characterized by a lower level of MDA with respect to p53^K120R^, p53^3KR^, and p53^R273H^ cells both in untreated and treated conditions (Figure 7).

The increment of cellular oxidative stress in presence of all type of P53 mutant proteins is evident despite an increase in expression of proteins involved in antioxidant response (i.e., G6PD, GCLC, GCLM and xCT) (Figure 8), suggesting that cells expressing P53 mutant proteins, especially the p53^3KR^, are able to sense the oxidant environment without being able to really counteract the oxidative stress, differently from p53^WT^ cells. In p53^3KR^ cells, the activation of an antioxidant response can also be inferred by the significant increase of G6PD, GCLC, GCLM and xCT not detected in other P53 mutant environment (Figure 8). Thus, the antioxidant response can be activated in p53^3KR^ carrying cells, but it does not go to completion.

We evaluated the activity of the p53^K120R^ mutant also in comparison to the human triple p53^3KR^ mutant. Li and others, by using a mouse model found that the mouse p53^3KR^ protein failed to activate P53 target genes of cell cycle arrest or apoptosis, but it retained the ability to regulate metabolism as p53^WT^ by repressing *xCT*, coding for the main transporter for cystine (Jiang et al., 2015; Liu et al., 2019; Liu and Gu, 2022, 2021; Xia et al., 2022). Our data, while confirming the ability of p53^K120R^ and the inability of p53^3KR^ mutant to induce P21 and MDM2 at protein level (Figure 2), show a significant induction of xCT protein level by p53^K120R^ and p53^3KR^ after the oxidative challenge (Figure 8).

The NRF2 transcription factor is one of the main regulators of the genes involved in the antioxidant response, including *xCT*. In breast cancer cells, the NRF2-dependent transcription of *xCT* can be inhibited by a direct interaction of NRF2 with mutant P53 proteins (Lisek et al., 2018); as a consequence, breast tumor cells with a P53 mutant protein may present low levels of *xCT* expression, resulting in low GSH and high ROS levels (Liu et al., 2017). In the HCT116^*TP53*−/−^ cellular background it was shown that also the wild type P53 protein suppresses the transcription of *xCT* by NRF2 (Faraonio et al., 2006). Taking these observations into consideration, we argue that in our experimental system neither wild type nor mutant P53 proteins interfere with NRF2, thus determining high levels of xCT protein. Moreover, NRF2 protein level did not change in any transfection and treatment conditions (Supplementary Figure S7), suggesting a NRF2-independent regulation of xCT in these experimental conditions.

Interestingly, we observe an induction of xCT in presence of p53^WT^ protein both in untreated and H_2_O_2_ treated cells. Although the repressing activity of P53 on SLC7A11 expression has been reported in U2OS and MCF7 human cell lines (Jiang et al., 2015), in HCT116^*TP53*−/−^ colon cancer cells P53 stimulates, instead of repressing, SLC7A11 expression. Our observation finds a confirmation in a recent paper by Xie et al. (2017), where p53^WT^ protein mediate xCT expression in HCT116^*TP53*−/−^ cells. In these cells, P53 wild type by interacting with the dipeptidyl-peptidase-4 (DDP4) protein, involved in lipid peroxidation, favors its nuclear localization; inside the nucleus it was supposed that DDP4 acts as P53 transcription cofactor, stimulating xCT expression (Xie et al., 2017). Since we observed an induction of xCT following H_2_O_2_ in HCT116^*TP53*−/−^ cells with different P53 backgrounds, we can hypothesize an involvement of this pathway in the modulation of the antioxidant response in HCT116 transfected cells. Thus, the activation of SLC7A11 by P53 can be considered tissue/cell line-dependent and not in contradiction with results obtained in other cell lines. This observation is in keeping with data reported in Eriksson et al., 2017 where cancer cell type specific metabolic functions of mutant P53 have been postulated (Eriksson et al., 2017). It has to be underlined that, even though we reported an increase of SLC7A11 protein following H_2_O_2_, the antioxidant response in mutant P53 carrying cells remains defective. Thus, our results indicate that, especially in the p53^3KR^ cells, an antioxidant response is activated with the tentative to balance the increment of oxidative stress, but this response is not sufficient to avoid the lipid peroxidation, as indicated by the MDA load.

The effect of expression of different types of P53 mutant proteins on glycolysis and mitochondrial metabolism has been previously addressed (Eriksson et al., 2017). By analyzing the metabolic features of HCT116 and H1299 cells transfected with different *TP53* hot spot mutations, metabolic heterogeneity was observed based on the type and the stability of mutant P53 proteins; in general, it was observed that the OxPhos activity resulted altered in presence of most P53 mutant proteins compared to the control cells, while the ECAR (Extra Cellular Acidification Rate), a surrogate measure of lactate export, appeared significantly enhanced, suggesting a metabolic switch to the anaerobic energy metabolism. Specifically, the inducible expression of p53^R237H^ caused the increment of both glycolytic capacity and OCR, in agreement with our data.

In conclusion, our results underline that not all P53 mutant proteins can be considered metabolically equivalent. We demonstrated that cells carrying p53^K120R^ are characterized by a metabolic phenotype that is intermediate between p53^WT^ and p53^R273H^ expressing cells, indicating a partial activity by the peculiar *TP53* K120R cancer mutation in modulation of cellular metabolism. On the other hand, p53^R273H^ cells showed the most evident metabolic change, which could compromise the cellular environment in agreement with its LoF and GoF activities. Such diversity can rely not only on P53 transcriptional specificity, as emerging from our results, but also from the interconnection with the cellular environment in which the P53 mutant protein can operate.

## Data Availability

The original contributions presented in the study are included in the article/Supplementary Material further inquiries can be directed to the corresponding author.
