# Erratum: Mutant p53K120R expression enables a partial capacity to modulate metabolism

**DOI:** 10.3389/fgene.2022.1088645

**Published:** 2022-11-16

**Authors:** Frontiers Production Office

**Affiliations:** Frontiers Media SA, Lausanne, Switzerland

**Keywords:** p53, energy metabolism, K120R mutation, antioxidant response, lipid peroxidation

Due to a production error, there was mistake in the **Affiliation** of author “Paola Monti.” The author is only affiliated to 1 “Mutagenesis and Cancer Prevention Unit, IRCCS Ospedale Policlinico San Martino, Genoa, Italy.”

The publisher apologizes for this mistake. The original version of this article has been updated.

