# Supplementary material for: Mutant p53K120R expression enables a partial capacity to modulate metabolism
Source: Front Genet. 2022 Sep 26;13:974662. doi: 10.3389/fgene.2022.974662 (PMC9549157; doi:10.3389/fgene.2022.974662)

Supplementary Material

Supplementary Fig. S1

Sequence alignment of human (NP_000537.3) and mouse (NP_035770.2) P53 proteins (α isoform: 393 and 390 amino acids, respectively)

Protein sequences were obtained from NCBI and aligned by using Align analysis tool on UniProt web site. Indicated are amino acids that show identity (*), strong similarity (:) or weak similarity (.), according to the alignment software. The positions of the human P53 K120, K164 and Q165 codons with the corresponding numbering of mouse P53 protein (K117, K161 and K162) are also indicated.

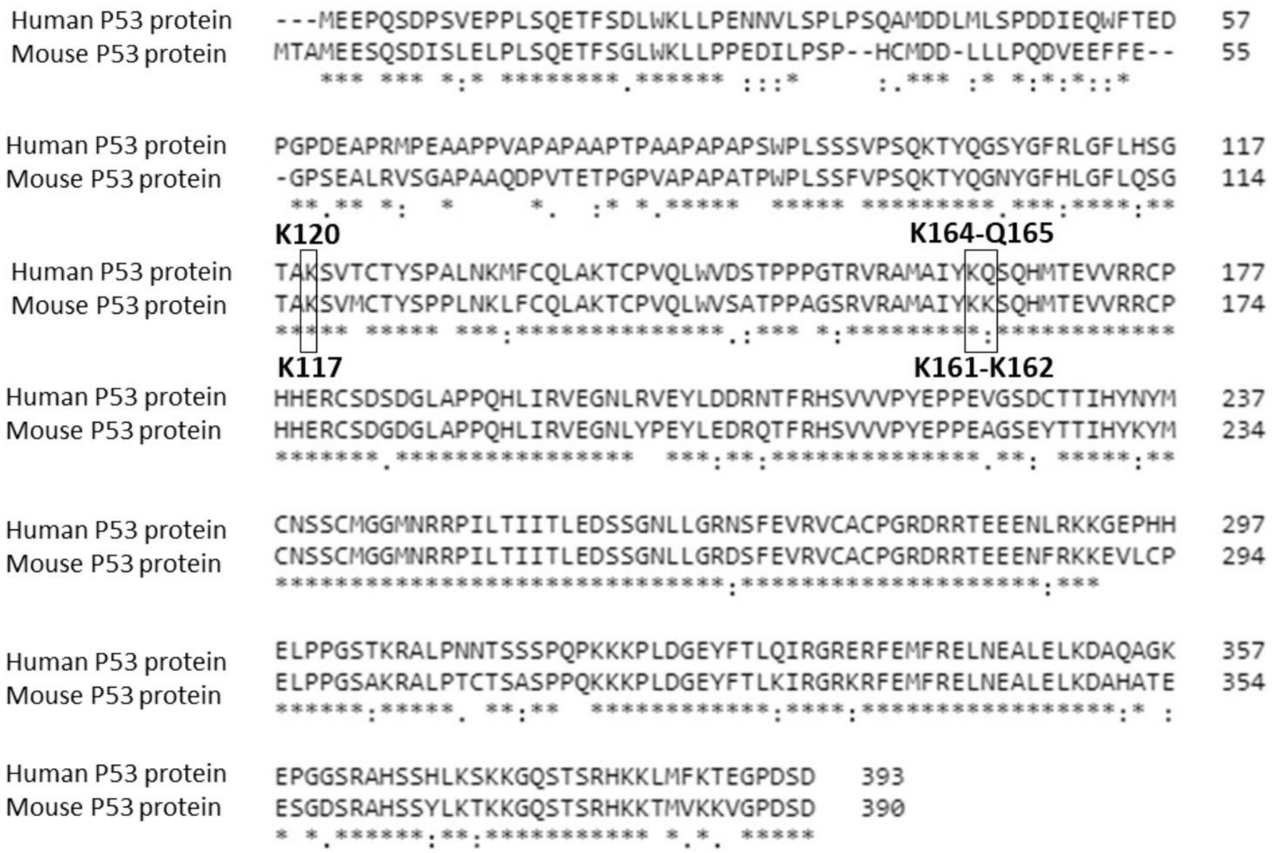

Supplementary Fig. S2

Percentage of living cells following H_2O_2 treatment in HCT116$^{TP53-/-}$ cells expressing p53^{WT} (WT), p53^{3KR} (3KR), p53^{K120R} (K120R) and p53^{R273H} (R273H) proteins.

HCT116$^{TP53-/-}$ cells were transiently transfected with the empty vector (empty) or plasmid expressing p53^{WT}, p53^{3KR}, p53^{K120R} and p53^{R273H}. After 16 hours of transfection, cells were treated with 100 μM H_2O_2 for further 6 hours. At the end of treatment, cells were trypsinized and counted; living cells were evaluated with trypan blue exclusion using a TC20 apparatus (Bio-Rad). As shown in the histogram, the percentage of living cells was comparable among samples. The same number of cells ($1x10^5$) was used to determine metabolic end points. Data were obtained from at least five independent experiments and reported as mean ± SD.

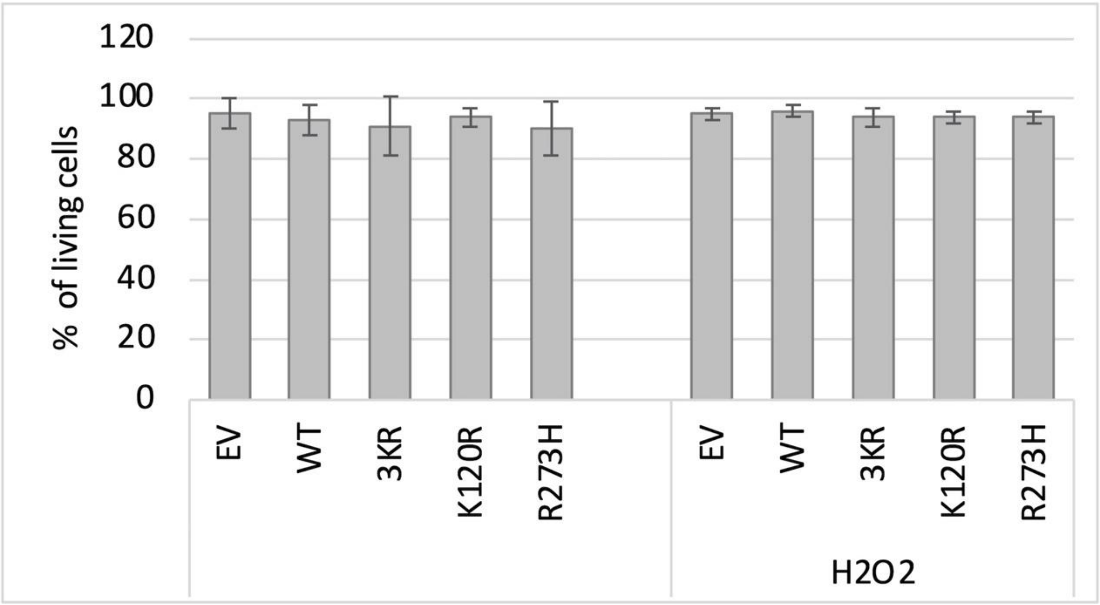

Supplementary Fig. S3

P53 proteins detected in $HCT116^{TP53-/-}$ cells transfected with $p53^{WT}$, $p53^{3KR}$, $p53^{K120R}$ and $p53^{R273H}$ expression vectors.

A) Representative western blots showing the level of P53 and beta-actin (β-act) in $HCT116^{TP53-/-}$ cells transiently transfected with the empty vector (EV), $p53^{WT}$, $p53^{3KR}$, $p53^{K120R}$, $p53^{R273H}$ and $p53^{2KR}$ expression plasmids. B) Histogram representing the amount of p53 protein detected in $HCT116^{TP53-/-}$ transfected cells and normalized for β-actin. Data were obtained after chemiluminescence analysis of western blots from at least four independent experiments and are reported as mean ± SD.

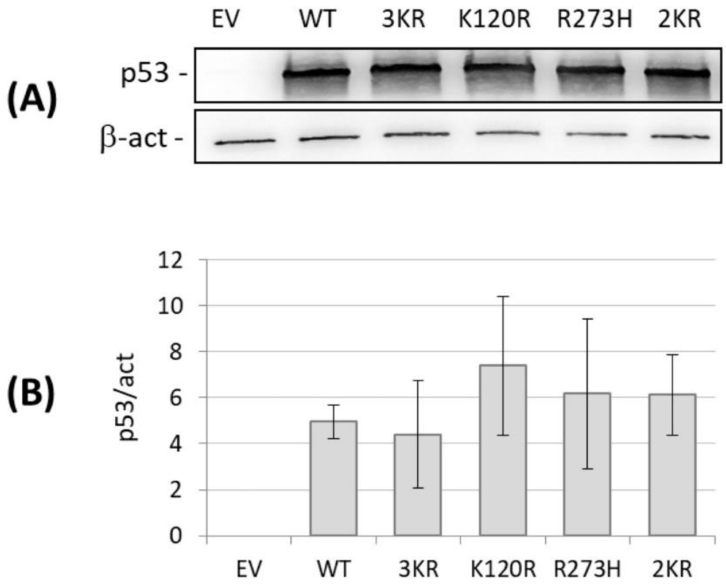

The expression level of different P53 protein in $HCT116^{TP53-/-}$ cells following transfection was comparable. A plasmid carrying the double P53 mutation K120R+K164R, was also constructed and expressed in $HCT116^{TP53-/-}$ cells. The expression level of the human $p53^{3KR}$ (K120R+K164R+ Q165R) carrying the Q165R amino acid substitution, did not significantly affect the formation of the P53 protein.

Supplementary Fig. S4

Inhibition of OCR and ATP synthesis by rotenone and antimycin A in HCT116$^{TP53-/-}$ cells expressing p53^{WT}, p53^{K120R}, p53^{3KR} and p53^{R273H} proteins.

Samples were treated with 1 µM Rotenone or 10 µM Antimycin A, specific inhibitors of Complex I and Complex III, respectively, to verify that OCR and ATP synthesis are dependent on OxPhos machinery. Oxygen consumption rate and ATP synthesis were analyzed as described in the Material and Methods section in the main text.
A) OCR induced by pyruvate + malate (P/M). B) ATP synthesis induced by P/M. C) OCR induced by succinate. D) ATP synthesis induced by succinate. Each panel is representative of at least four independent experiments; data are reported as mean ± SD. The symbol **** indicates significant differences for $p<0.0001$ between untreated and rotenone- or antimycin A-treated samples.

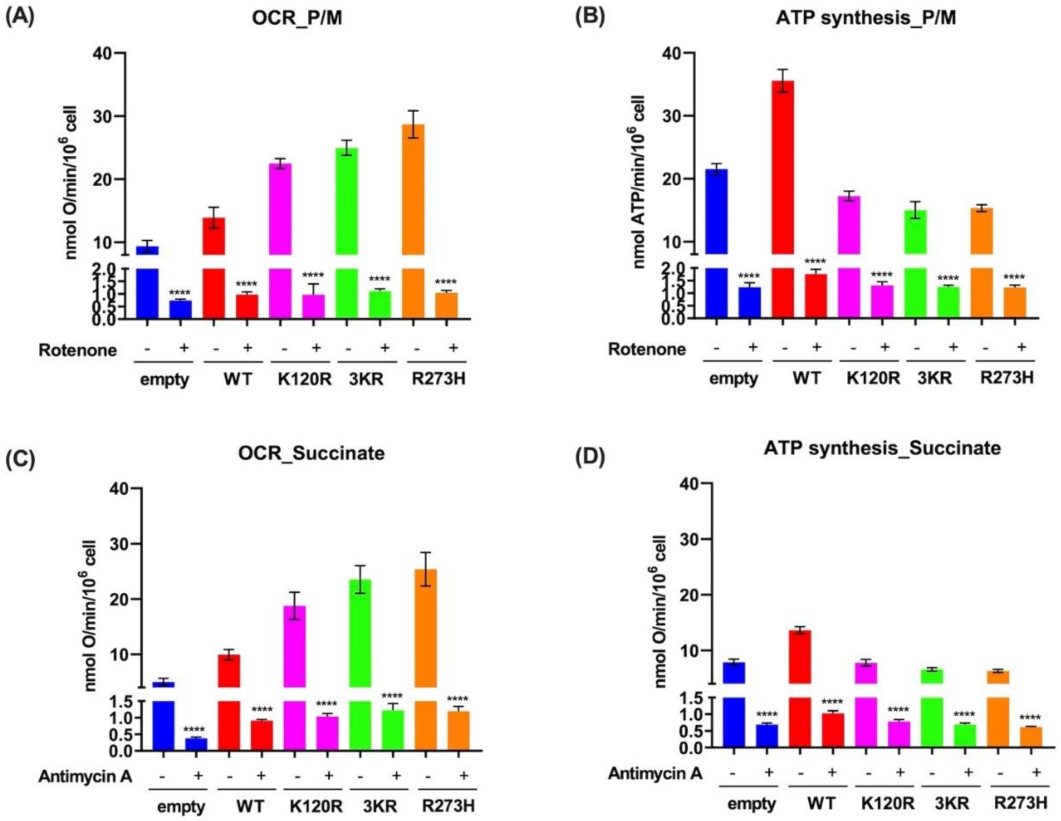

GLUT1 expression in HCT116$^{TP53-/-}$ cells expressing p53^{WT}, p53^{3KR}, p53^{K120R} and p53^{R273H} proteins.

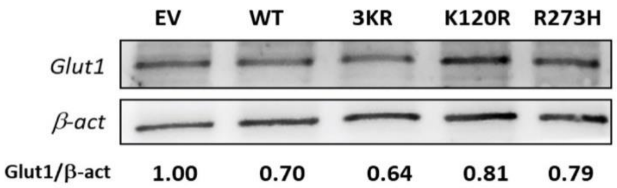

Western blots showing the level of GLUT1 and β-actin (β-act) in untreated HCT116$^{TP53-/-}$ cells transiently expressing p53^{WT}, p53^{3KR}, p53^{K120R} and p53^{R273H}. The expression level of GLUT1

normalized for β-actin is reported as Glut1/β-act ratio after chemiluminescence analysis of the membrane by UVITEC (Cambridge, UK). With respect to cells transfected with the empty vector (EV), all samples showed a slightly lower level of GLUT1, as expected for an inhibitory effect of P53 on GLUT1 expression. However, this analysis did not reveal differences among cells expressing wild type or mutant P53 protein.

Supplementary Fig. S5

Glutamine and beta oxidation metabolism in HCT116$^{TP53-/-}$ cells expressing p53^{WT}, p53^{K120R}, p53^{3KR} and p53^{R273H} proteins.

A) Glutaminase (GLS) activity. B) Glutamate dehydrogenase (GDH) activity. C) 3-hydroxyacyl-CoA dehydrogenase activity. Activities reported in panels A and B are markers of aminoacidic metabolism; the activity reported in panel C is a marker of fatty acids beta-oxidation. Each panel is representative of at least four independent experiments; data are reported as mean ± SD. The symbols *, **, and **** indicate significant differences for $p<0.05$, 0.01, and 0.0001, respectively, between empty and the other samples; the symbol #, ##, and #### indicates significant differences for $p<0.05$, 0.001, and 0.0001, respectively, between p53^{K120R} and the indicated samples.

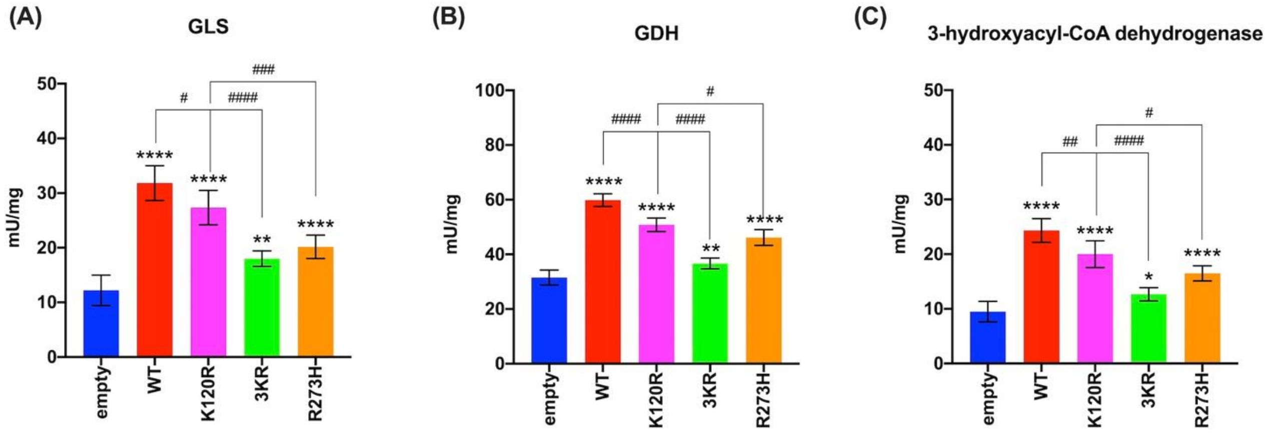

Supplementary Materials & Methods

Glutaminase and glutamic dehydrogenase activity assay

Glutaminase activity was assayed spectrophotometrically at 340 nm following NAD$^+$ reduction. The assay mix contained: Tris-HCl (pH 8), 50 mM glutamine, 5 mM NAD$^+$, and 5 IU glutamic dehydrogenase (1). Glutamic dehydrogenase was assayed spectrophotometrically at 340 nm following NADH oxidation. The assay solution contained: Tris-HCl (pH 7.4), 20 mM α-ketoglutarate, 0.15 mM NADH, and 1 mM ADP (1). In both cases, 0.1 mM rotenone was added to inhibit the NADH oxidation from Complex I. 50 μg of total protein were used for both assays and data were normalized on the sample protein content.

Fatty acid metabolism evaluation

The activity of 3-hydroxyacyl-CoA dehydrogenase, used as a marker of fatty acids beta-oxidation metabolism, was assayed spectrophotometrically at 340 nm following the oxidation of NADH in the presence of acetoacetyl-CoA. The reaction mix contained: 100 mM sodium phosphate (pH 6.0), 0.2 mM NADH and 0.1 mM acetoacetyl-CoA (2).

Supplementary References

1. Cappelli E, Cuccarolo P, Stroppiana G, Miano M, Bottega R, Cossu V, Degan P, Ravera S. Defects in mitochondrial energetic function compels Fanconi Anaemia cells to glycolytic metabolism. *Biochim Biophys Acta - Mol Basis Dis* (2017) **1863**:1214–1221. doi:10.1016/j.bbadis.2017.03.008
2. Cappelli E, Degan P, Bruno S, Pierri F, Miano M, Raggi F, Farruggia P, Mecucci C, Crescenzi B, Naim V, et al. The passage from bone marrow niche to bloodstream triggers the metabolic impairment in Fanconi Anemia mononuclear cells. *Redox Biol* (2020) **36**:101618. doi:10.1016/j.redox.2020.101618

Supplementary Fig. S6

Oxidative stress status in HCT116$^{TP53-/-}$ cells expressing p53^{WT}, p53^{K120R}, p53^{3KR} and p53^{R273H} proteins.

A) Evaluation of ROS production in p53^{WT}, p53^{3KR}, p53^{K120R}, and p53^{R273H} expressing cells; the symbols *, *** and **** indicate significant differences for p=0.0245, p=0.0001 and p<0.0001, respectively, between empty and the other samples. B) Intracellular level of GSH. The symbols ** and **** indicate significant differences for p≤0.0082 and p<0.0001, respectively, between empty and the other samples. Data are representative of at least four independent experiments and are reported as mean±SD. C) ROS/GSH values in the same transfected cells; the symbols **** indicate significant differences for p<0.0001 between empty and the other samples; the symbols ### and #### indicate significant differences for p < 0.0001 (p53^{K120R} vs and p53^{WT} and p53^{R273H}; p=0.002, p53^{K120R} vs p53^{3KR}

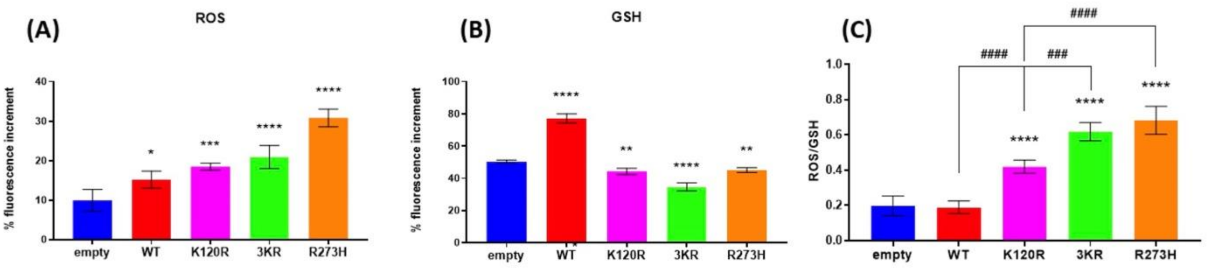

Supplementary Materials & Methods

ROS and GSH evaluation

To evaluate the reactive oxygen species (ROS) level, cells were washed and re-suspended in PBS and stained for 10 minutes at 37° C with 20,70-dichlorodihydro-fluorescein diacetate (H_2DCFDA) at a concentration of 5 µM (Thermo Fisher Scientific, Waltham, MA, USA). H_2DCFDA is a non-fluorescent dye, which is cleaved inside cells to 20,70-dichlorofluorescein (H_2DCF). In the presence of oxidants, H_2DFC is converted in turn to the fluorescent compound DCF. Samples were measured on a FacsCalibur flow cytometer (Becton Dickinson, San José, CA). The analysis was confined to viable cells only, after gating based on forward- and side-scatter characteristics. Ten thousand cells per sample were analyzed (Ravera et al., 2020, PMID: 33020573).

Glutathione (GSH) was measured by flow cytometry after staining cells with monobromobimane (MBB Thermo Fisher Scientific, Italy) (Cuccarolo et al., 2012, PMID: 22578062). When excited at 405 nm, MBB becomes fluorescent after conjugation with intracellular thiols. This reaction is far more competitive for free GSH than for protein sulfhydryls. The decrease in the intracellular GSH concentration is reflected by a decrease in the MBB fluorescence peak.

Supplementary Fig. S7

Modulation of NRF2 protein by p53^{WT}, p53^{3KR}, p53^{K120R}, and p53^{R273H} proteins in untreated and H_2O_2 treated HCT116$^{TP53-/-}$ cells.

A) Representative western blots showing the level of NRF2 and β-actin (β-act) endogenous proteins in untreated and 100 μM H_2O_2 treated HCT116$^{TP53-/-}$ cells transiently expressing p53^{WT}, p53^{K120R}, p53^{3KR}, and p53^{R273H}. B**)** Histogram representing the amount of NRF2 protein detected in HCT116$^{TP53-/-}$ transfected cells in untreated or treated samples and normalized for β-actin. Data were obtained after chemiluminescence analysis of western blots from at least two independent experiments and are reported as mean ± SD. The levels of the different proteins were calculated as fold over the level of the same proteins found in cells transfected with the empty vector; the dotted line corresponds to one-fold over empty.

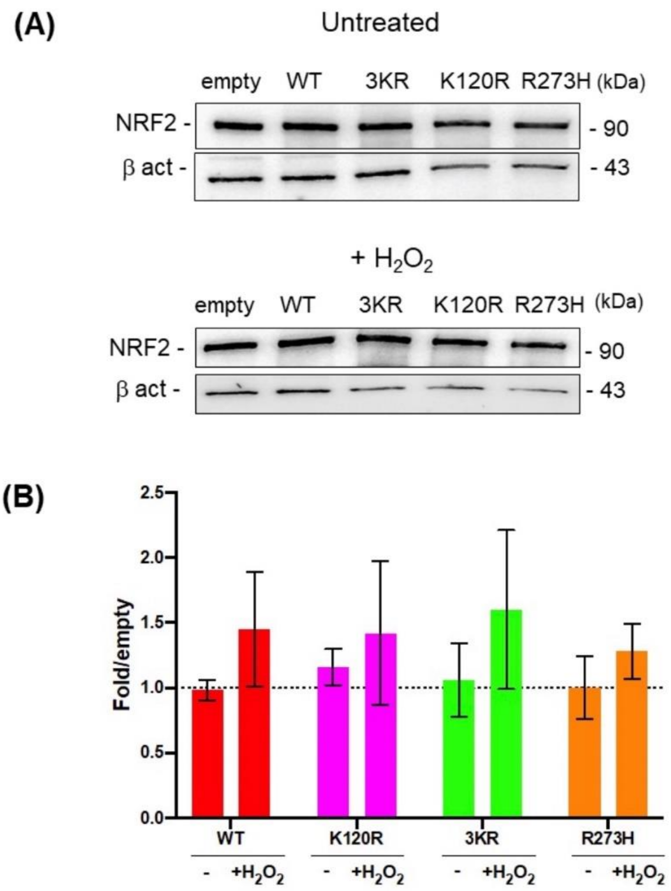

Supplementary Fig. S8

GPX4 modulation in HCT116$^{TP53-/-}$ cells expressing p53^{WT}, p53^{K120R}, p533KR and p53^{R273H} proteins.

A) Representative western blots showing the level of GPX4 and beta-actin (β-act) in HCT116$^{TP53-/-}$ cells transiently transfected with the empty vector (EV), p53^{WT}, p53^{K120R}, p53^{3KR}, and p53^{R273H} with or without H_2O_2 treatment. B) Histogram representing the amount of P53 protein detected in HCT116$^{TP53-/-}$ transfected cells, normalized for β-actin and reported as Fold/EV. Data were obtained after chemiluminescence analysis of western blots from two independent experiments and are reported as mean ± SD (Note that the K120R and 3KR samples in untreated and H_2O_2 treated western blot are loaded in a different order).

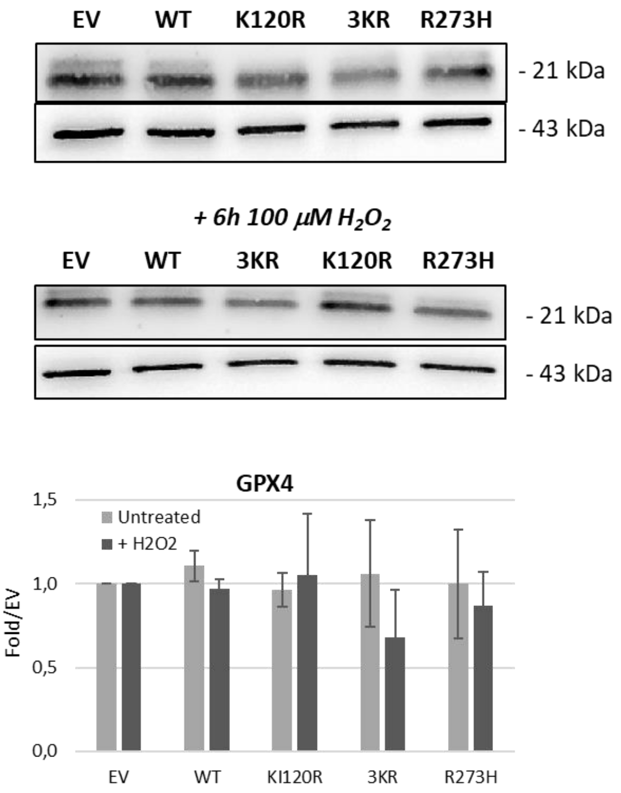

Supplement: Supplementary file 1 [file DataSheet1.pdf]
